# Holistic Health Approaches for People With Alcohol Use Disorder: Protocol for a Scoping Review

**DOI:** 10.2196/85036

**Published:** 2026-07-15

**Authors:** Shubhi Nanda, Anna E Roberts, Jenny Yarmovsky, Gisela Butera, Donna Barnett, Lorenzo Leggio, Gwenyth R Wallen, Jennifer J Barb

**Affiliations:** 1 Translational Biobehavioral and Health Promotion Branch, Clinical Center, National Institutes of Health, National Institutes of Health Clinical Center Building 10, Room 2B13, 10 Center Drive, Bethesda, MD, 20814, United States, 1 202- 961-1026; 2 Division of Library Services, NIH Library, National Institutes of Health, Bethesda, MD, United States; 3 Nutrition Department, Clinical Center, National Institutes of Health, Bethesda, MD, United States; 4 Clinical Psychoneuroendocrinology and Neuropsychopharmacology Section, Translational Addiction Medicine Branch, National Institute on Drug Abuse, Baltimore, MD, United States; 5 Center for Alcohol and Addiction Studies, Department of Behavioral and Social Sciences, Brown University, Providence, RI, United States; 6 Division of Addiction Medicine, School of Medicine, Johns Hopkins University, Baltimore, MD, United States; 7 Department of Neuroscience, Georgetown University Medical Center, Washington, DC, United States

**Keywords:** alcohol use disorder, AUD, holistic interventions, complementary therapies, scoping review, AUD treatment

## Abstract

**Background:**

Alcohol use disorder (AUD) is a complex, chronic medical disease wherein patients have traditionally benefited from a multidisciplinary and comprehensive therapeutic approach, including a comprehensive treatment plan that addresses the psychological, behavioral, and social dimensions of the individual. Holistic health is a nontraditional system of wellness that focuses on well-being through body, mind, and spirit and aims to restore balance through combined physical, mental, and emotional care. This approach may incorporate nonpharmacological and alternative treatments alongside allopathic medicine. In recent years, there has been a growing interest in integrating holistic health strategies into addiction treatment.

**Objective:**

The primary objective of this scoping review is to identify and characterize holistic health interventions currently used in the management of people with AUD. A secondary objective is to collate the reported efficacy of these interventions. Additionally, this review will identify gaps in the literature and suggest areas for future research.

**Methods:**

This review will adhere to the Arksey and O’Malley methodological framework for scoping reviews. Published research and pilot clinical trials reporting on holistic health interventions in AUD will be included. Five databases will be searched: PubMed and MEDLINE, Embase, CINAHL, Web of Science, and Scopus. There will be no date restrictions, though only English-language articles will be included. To qualify, studies must include at least one holistic intervention (eg, meditation, homeopathy, and special diets). Excluded studies will include those that do not involve people with AUD and/or hazardous drinking or that only test pharmaceutical treatments without a holistic component. Two reviewers will independently screen and extract data with a third reviewer as a tiebreaker; discrepancies will be resolved through discussion.

**Results:**

This scoping review began in Covidence in December 2024 and is currently funded by the National Institutes of Health Intramural Research Program. Data collection was completed in February 2026, and data analysis was completed in June 2026. Results are expected to be published in late 2026.

**Conclusions:**

This review will provide an overview of holistic approaches and interventions used in AUD treatment. It will highlight gaps in the literature and recommend directions for future research. Results will be submitted for publication in a peer-reviewed journal. No ethics approval is required because the review involves only publicly available literature.

## Introduction

### Background

Alcohol use disorder (AUD) is characterized by excessive alcohol consumption and related consequences and is identified by the *Diagnostic and Statistical Manual of Mental Disorders *as a chronic medical condition associated with significant impairment in social, personal, and occupational functioning [1]. AUD is prevalent in the United States, contributing to public health concerns such as accidents, violence, and long-term chronic health problems, which place a significant economic burden on the health care system [1,2]. Mutual support group (eg, Alcoholics Anonymous and SMART Recovery) engagement is associated with recovery from AUD [3]. Furthermore, while underutilized, conventional therapeutic approaches include brief interventions and evidence-based behavioral (eg, cognitive behavioral therapy [CBT] and motivational interviewing [MI] or motivational enhancement therapy) and/or pharmacotherapeutic treatments [4]. Standard treatment models frequently emphasize pharmacologic management; however, psychosocial therapies remain central to evidence-based care.

In parallel with these established treatments, there is growing interest in integrative and holistic health approaches that emphasize the interconnectedness of mental, physical, and emotional well-being [5-7]. These approaches are conceptualized not as replacements for conventional care but as adjunctive or complementary strategies that may enhance recovery outcomes in individuals with AUD [8].

Culturally and linguistically diverse populations tend to have high rates of addictive disorders and lower rates of treatment seeking and treatment completion than the general population. The lack of culturally relevant and appropriate treatment presents a significant barrier for these patient populations [9]. This highlights the importance, from both a scientific and public health perspective, of considering personal characteristics (eg, race and ethnicity) and contextual factors (eg, socioeconomic conditions and environmental stressors) in treatment. There remains a limited understanding of how clinicians’ cultural competence can be strengthened to better support these populations [10]. Integrative and holistic practices may enhance culturally responsive care by allowing treatment to be tailored to individual beliefs, values, and lived experiences [10,11].

Holistic health, in this review, refers to an integrative, whole-person framework that addresses biological, psychological, behavioral, and sociocultural dimensions of health. This approach may incorporate various practices, including mindfulness, nutrition therapy, exercise, psychotherapy, acupuncture, yoga, meditation, breathing exercises, herbal remedies, social support, spirituality and religion, and stress management techniques. By targeting multiple interconnected contributors to alcohol use—including stress, trauma exposure, mental health comorbidities, and lifestyle factors—holistic approaches aim to promote sustained recovery rather than solely symptom reduction. CBT and MI, which focus on modifying thoughts, behaviors, and motivation, are included within this multidimensional framework, as they address psychological and behavioral determinants of alcohol use [12-14]. Despite growing interest in these practices, the research on how holistic interventions can be integrated into AUD treatment remains limited. This gap is furthered by limited training in culturally responsive substance use care, showcasing the need to better characterize holistic approaches that extend beyond conventional approaches [10].

### Study Rationale

In the context of AUD, the evidence base is fragmented and methodologically diverse. Interventions span multiple disciplines, including psychology, nutrition, complementary and alternative medicine, and integrative health, and use a wide range of outcome measures, from abstinence and relapse rates to mental health and quality-of-life indicators. To date, no comprehensive effort has been made to map this landscape systematically. A scoping review is therefore warranted to collate and categorize the breadth of interventions, synthesize reported outcomes, and identify areas of concentrated versus limited evidence. This mapping will provide an essential foundation for future systematic reviews or meta-analyses, as well as prospective studies targeting specific intervention types, while also guiding clinicians, researchers, and policymakers in understanding the current state of the science on holistic approaches to AUD treatment. Holistic and integrative strategies aim to complement standard care by addressing these multidimensional determinants of health.

Although behavioral therapies such as CBT and MI are considered standard psychosocial care, limited research examines their structured integration with complementary modalities such as mindfulness-based interventions, nutritional therapy, or lifestyle modification programs. These combined approaches may enhance recovery outcomes by supporting both symptom stabilization and broader well-being [12]. Additionally, sociocultural influences—such as family dynamics and cultural norms surrounding alcohol use—are pivotal in both the development and recovery from AUD. However, they remain underrepresented in the conventional treatment paradigm [15].

Research on holistic and integrative interventions for AUD has emerged across multiple domains, including nutritional therapies. For example, a 2023 study examining the effects of dietary intake on the gut microbiome of individuals with AUD investigated the impact of fiber intake [16]. The study found that a fiber-rich diet helped curb alcohol cravings during withdrawal and may aid in maintaining abstinence [16]. This research suggests that diet may influence mood and alcohol cravings, highlighting the potential for nutritional interventions as part of a holistic treatment plan [17]. However, this body of work remains fragmented, with limited efforts to systematically map or categorize the range of approaches and reported outcomes.

### Study Objectives

This review aims to understand the current literature by synthesizing existing research on the use of holistic practices in the treatment of AUD. In this context, “holistic” refers to nonpharmacologic or adjunctive interventions that address biological, psychological, behavioral, and sociocultural dimensions of health and may be delivered alongside standard psychosocial and conventional care. Specifically, it will examine a range of treatment modalities, including mind-body interventions, nutritional therapies, and psychosocial approaches, to provide a more comprehensive perspective on AUD recovery. This scoping review also aims to provide a descriptive mapping of reported outcomes rather than a formal synthesis of intervention effectiveness, allowing for a better understanding of the types of holistic interventions found. The review will also examine how these holistic strategies can be integrated with conventional treatments, aiming to improve both immediate recovery outcomes and long-term well-being. Ultimately, this work aims to advance the understanding of the role that holistic health practices may play in supporting sustained recovery from AUD. The protocol for this review was preregistered [18], and also a preprint is available [19].

## Methods

### Protocol Design

A scoping review will be conducted to provide an overview of the presence of holistic interventions, their characteristics, and key factors of implementation as reported in the supporting literature. The review will follow the methodological framework developed by Arksey and O’Malley [20] and updated by Joanna Briggs Institute methodology for scoping reviews and the PRISMA-ScR (Preferred Reporting Items for Systematic Reviews and Meta-Analyses extension for Scoping Reviews) guidelines [20-22]. The PRISMA-ScR guidelines and checklist for scoping reviews are used to report the completed review (Checklist 1). The 5 stages of the methodological framework for scoping reviews developed by Arksey and O’Malley [20,23] will be used.

### Research Question

The overarching aim of this review is to synthesize existing research on the use of holistic practices in the treatment of AUD, as well as highlight corresponding gaps in the literature. The research team defined the following four research questions:

1. What holistic interventions are currently being implemented in the treatment of AUD, and how do these interventions impact AUD recovery outcomes?
2. Which holistic interventions have been used more commonly in treating AUD?
3. What are the perceived barriers to implementing holistic approaches in AUD treatment?

The following databases will be searched: PubMed and MEDLINE (National Library of Medicine), Embase (Elsevier), CINAHL Plus (EBSCOhost), Web of Science Core Collection (Clarivate), and Scopus (Elsevier).

### Eligibility Criteria

To develop our search strategy, the 3-step approach outlined in the Joanna Briggs Institute Scoping Review Guidance will be applied [24]. The search strategy will be developed using relevant Medical Subject Headings (MeSH) terms and additional keywords related to AUD and various holistic health interventions. For a full list of search terms, refer to Multimedia Appendix 1. The search strategy will be created in collaboration with the National Institutes of Health (NIH) Library to ensure alignment with the objectives of this scoping review. Searches will be undertaken from database inception, with no restrictions on publication date. Currently, 2 searches are being conducted: one that searches all publications up to November 2024 and another that searches from November 2024 through September 2025, using the MeSH terms developed to include as many potential studies in this scoping review as possible. All references retrieved from the searches will be exported to EndNote (version 21; Clarivate), where duplicates will be identified and removed. The search strategy aims to locate both published and unpublished studies. We will include primary studies using any methodology (including experimental, quasi-experimental, observational, mixed methods, and qualitative study designs) that explore the use of holistic health interventions in treating AUD. Case reports, editorials, letters, or opinion pieces will not be included. Additionally, we will perform backward and forward searching of the reference lists of included papers to identify any additional relevant studies.

### Study Selection

Following the database searches, all identified citations will be transferred to Covidence (Veritas Health Innovation), a screening software, and additional duplicates will be excluded [25]. The titles and abstracts will be independently screened against the eligibility criteria by 3 researchers (SN, AER, and JY; Textbox 1). The full texts of all potentially eligible articles will be retrieved, read in full, and assessed against the eligibility criteria. Title and abstract screening and full-text assessment will be completed by 3 members of the review team. Any discrepancies regarding the eligibility of an article at any stage will be resolved through discussion, and if necessary, an additional reviewer will be involved. If eligibility remains unclear, these discussions can be extended to the full review team. We will produce a PRISMA (Preferred Reporting Items for Systematic Reviews and Meta-Analyses) flowchart of the study inclusion process, recording the number of articles retained at each screening stage and the reasons for exclusion at the full-text screening stage [22]. Covidence will be used to store and organize all articles. We will also record, tabulate, and report papers not available in English but that otherwise meet our eligibility criteria.

Textbox 1. **Inclusion and exclusion criteria for determining review selection.**

**Inclusion**

- Human participants
- Patient population diagnosed with alcohol use disorder (AUD) or population that exhibits drinking patterns that clearly align with the diagnostic criteria for AUD (alcohol misuse and hazardous drinking)
- At least one holistic health intervention (complementary and integrative interventions that address biological, psychological, and sociocultural dimensions of health)
- Patient population was treated in a clinical, community, inpatient, or outpatient setting

**Exclusion**

- Nonhuman participants
- Participants with nonalcoholic liver disease or that exhibit normal drinking behavior or drinking behavior not considered to be alcohol misuse, AUD, or hazardous drinking
- Interventions that do not include a holistic health component

### Data Charting

Before the data extraction phase, the team will need to confirm access to all publications and, if possible, gain access to publications that are unavailable through the NIH Interlibrary Loan system. If the full text of an original paper is unavailable, we will contact the corresponding authors of included articles to obtain the full text as well as information that is either missing or unclear.

Through Covidence, a custom template for data extraction will be used to extract data as it appears in the full-text review. Interrater reliability will be assessed through a 3-reviewer method, where 2 reviewers extract the data, and a third reviewer will compare the extracted data. If there is agreement among the reviewers regarding the papers and the extracted data, the studies will be divided among all reviewers. If any issues arise with the review process, the team will meet to discuss and re-evaluate it to ensure reliability. The data extraction, which will be charted as part of this scoping review, is presented in Table 1.

**Table 1.** Detailed information to be included and extracted in the literature review.

Category	Description
Article information	Link to full text Paper number Title Year published Journal in which the paper was published Data collection location (country, state, and city)
Participant characteristics	Alcohol-associated diagnosis (alcohol use disorder, alcohol-associated liver disease, cirrhosis, etc) Diagnostic tool for the alcohol-associated diagnosis Sample size Sex: male and female participants (n, %) Age (mean, SD or median, range) Race or ethnicity Employment status BMI and weight (mean, SD or median, range)
Study characteristics and outcomes	Study design (cross-sectional, longitudinal, etc) Pharmaceutical health intervention used (yes or no) Holistic health intervention used Pharmaceutical intervention used (if applicable) Collection limitations Important notes Limitations of the study Type of data collected (qualitative or quantitative)

### Data Synthesis

The holistic strategies identified will be compiled and presented from the included research to characterize the current approaches to managing and treating AUD (Table 1). Each intervention’s description, goal, and frequency of use will be included in this table. Additionally, we will look at and report on any assessment or outcome measures that are used to gage how beneficial these interventions are, as well as the frequency with which they are reported across studies.

The following main objectives will be considered and reported:

- Which holistic approaches have been applied to the treatment or management of AUD in the literature?
- What justification, if any, is given for the selection of holistic therapies for the treatment of AUD?

Additionally, to understand any gaps in the literature, members of the review team will separately tabulate and report on participant characteristics for all studies included in the review as part of the data collection.

The following questions will be discussed in this review of the data extracted:

- What details on the participants are included in the studies?
- Are socially stratifying elements (such as sex, race, and contextual factors, including environmental exposures and socioeconomic conditions) considered and reported?
- What justification, if any, is given for the selection of the study participant groups?

The results will be summarized in text, presented in tables for clarity, and published as multimedia appendices. The results will be categorized thematically, with holistic and integrative interventions grouped into broad conceptual categories (eg, mind-body, nutritional, psychosocial, and culturally grounded approaches) rather than subjected to formal qualitative analysis. A narrative summary of the identified interventions, outcome measures, and participant characteristics will be provided, including justifications for the choices made in the studies and any information that was not reported. Any implications of these findings will be discussed in the Discussion section of the report. A quality appraisal of the included studies will not be conducted as this is a scoping review designed to assess the current literature rather than study quality.

### Ethical Considerations

This scoping review does not require an ethical review or approval because it involves the collection and review of material that is publicly available. The review will be submitted for publication and is registered with the Open Science Framework. The findings of this review will help other researchers identify and establish current gaps in the use of holistic interventions for people with AUD. The research team will not need patient or public involvement for the proposed scoping review.

## Results

**Figure 1.**
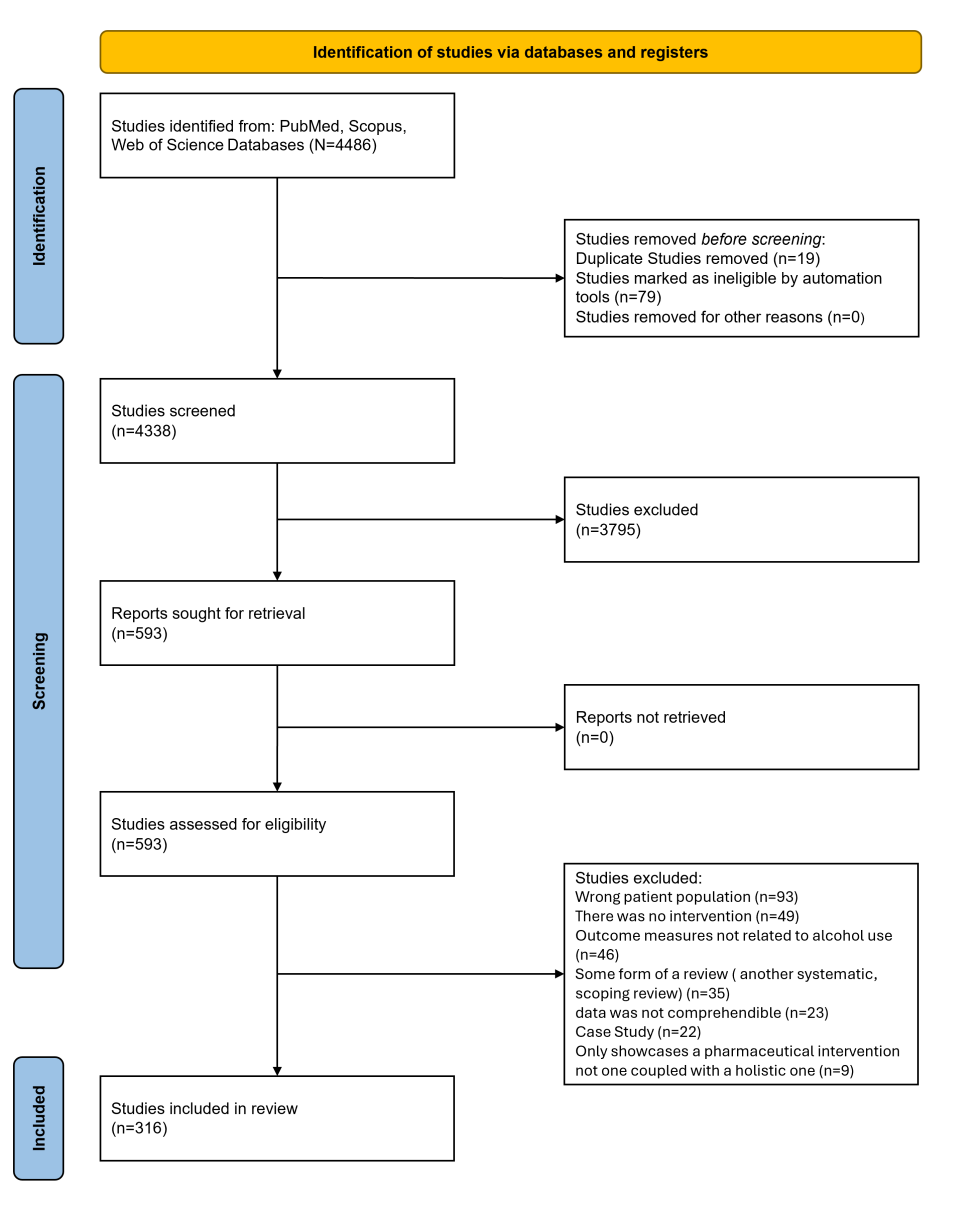
PRISMA-ScR (Preferred Reporting Items for Systematic Reviews and Meta-Analyses extension for Scoping Reviews) flowchart for the scoping review and current progress.

This scoping review was initiated in Covidence with an initial pilot performed in December 2024, and the full review screening began in January 2025 and is currently funded by the NIH Intramural Research Program. All the database searches were completed and then imported into Covidence for data collection and in total had produced 4486 studies. Of these, 19 (0.4%) duplicates were identified manually and 79 (1.8%) duplicates were identified by Covidence, resulting in 98 (2.2%) duplicates removed in total. Title and abstract screening of the remaining 4388 (97.8%) articles as well as full-text screening for eligibility is currently ongoing. Data collection was completed in April 2026; data analysis was completed in June 2026. The results of this review will be presented in a final manuscript related to the aims of this scoping review. Figure 1 presents a PRISMA flow diagram indicating the database searches. The final manuscript for this scoping review will include a narrative description of the findings and an extraction table.

## Discussion

### Anticipated Findings

This scoping review is anticipated to synthesize the existing literature examining holistic and integrative interventions used in the treatment of AUD because the existing evidence appears to vary substantially in intervention type, theoretical framing, outcome measures, and study design. While some studies suggest that adjunctive holistic approaches may improve treatment engagement, symptom burden, or recovery-related outcomes, others report mixed or inconclusive findings. We expect that mapping the literature will highlight considerable variability in how holistic interventions are defined, implemented, and evaluated, which likely contributes to the inconsistency observed across studies.

By consolidating and categorizing this body of evidence, this scoping review aims to clarify methodological patterns, identify conceptual reporting strengths and gaps, and characterize how holistic interventions have been integrated alongside standard care for AUD. This work will provide a comprehensive overview of the range of approaches studied, the outcomes assessed, and the populations represented, thereby informing future research directions. By doing so, this review will help identify areas where more rigorous evaluation may be warranted.

### Strengths and Limitations

This study has several strengths and limitations. A framework is outlined to extract information on holistic health interventions used for the treatment of AUD while assessing the efficacy of the approaches identified in this review. This review will also collate treatments that are used in concert with evidence-based conventional approaches for the treatment of AUD. The scoping review will be conducted using established methodological frameworks, including the Arksey and O’Malley framework [20] and the PRISMA-ScR checklist, to ensure transparency and reproducibility of the process. The aim is to synthesize a wide range of interventions using diverse MeSH to better characterize the development of these interventions in a heterogeneous population. A standardized data extraction form will be used to systematically capture and synthesize relevant study characteristics and outcomes across sources, supporting structured summarization by the review team. The reviewers represent a range of perspectives and academic backgrounds, which will help to address any bias in the interpretation of the reviewed studies. However, due to resource and reviewer language constraints, only English-language articles will be included, which may limit the generalizability and global applicability of the review findings. While the review is limited to English-language articles, included studies frequently focus on non–English-speaking and culturally diverse populations, indicating that publication language does not restrict population inclusion. Reporting of participant characteristics varied substantially across studies. While age and sex were commonly reported, race, ethnicity, and socioeconomic indicators were inconsistently described, limiting assessment of representation across equity-relevant domains and making comprehensive extraction infeasible without substantial missing data. Although nonindexed practitioner reports and organizational publications were explored, most did not meet the eligibility criteria, reflecting limited standardized reporting of holistic and integrative AUD interventions outside the peer-reviewed literature.

### Conclusions

Our protocol for a scoping review to synthesize the current literature focused on the use of holistic practices in the treatment of AUD is presented. The scoping review will comprehensively analyze existing research on holistic interventions, such as mind-body therapies, nutritional strategies, and psychosocial support, as well as evaluate if and how these approaches are integrated with conventional care, and identify gaps in the literature to guide future research. Synthesizing currently available literature to understand the state of the science has the potential to stimulate future hypotheses regarding the efficacy and implementation of holistic strategies in AUD recovery. Gaining a deeper understanding of how holistic health practices impact recovery outcomes may inform more comprehensive, person-centered treatment approaches and support sustained abstinence and well-being in individuals with AUD.

## Supplementary material

10.2196/85036 Multimedia Appendix 1 Medical Subject Headings (MeSH) terms used for search strategy in example database.

10.2196/85036 Checklist 1 PRISMA-ScR checklist.

## Abbreviations

AUD: alcohol use disorder
CBT: cognitive behavioral therapy
MeSH: Medical Subject Headings
MI: motivational interviewing
NIH: National Institutes of Health
PRISMA: Preferred Reporting Items for Systematic Review and Meta-Analyses
PRISMA-ScR: Preferred Reporting Items for Systematic Reviews and Meta-Analyses extension for Scoping Reviews
